# Vascular Dysfunction in Patients with Chronic Arsenosis Can Be Reversed by Reduction of Arsenic Exposure

**DOI:** 10.1289/ehp.7471

**Published:** 2004-12-09

**Authors:** Jingbo Pi, Hiroshi Yamauchi, Guifan Sun, Takahiko Yoshida, Hiroyuki Aikawa, Wataru Fujimoto, Hiroyasu Iso, Renzhe Cui, Michael P. Waalkes, Yoshito Kumagai

**Affiliations:** ^1^Graduate School of Medical Sciences, University of Tsukuba, Tsukuba, Japan; ^2^Laboratory of Comparative Carcinogenesis, National Cancer Institute, National Institutes of Health, Department of Health and Human Services, Research Triangle Park, North Carolina, USA; ^3^Department of Preventive Medicine, St. Marianna University School of Medicine, Kawasaki, Japan; ^4^Department of Labor Hygiene and Occupational Health, School of Public Health, China Medical University, Shenyang, China; ^5^Department of Public Health, Asahikawa Medical College, Asahikawa, Japan; ^6^Department of Environmental Health, Tokai University School of Medicine, Isehara, Japan; ^7^Department of Dermatology, Kawasaki Medical School, Kurashiki, Japan; ^8^Department of Public Health, and; ^9^Department of Environmental Medicine, Graduate School of Comprehensive Human Sciences, University of Tsukuba, Tsukuba, Japan

**Keywords:** arsenic, cGMP, endothelial dysfunction, intervention study, nitric oxide

## Abstract

Chronic arsenic exposure causes vascular diseases associated with systematic dysfunction of endogenous nitric oxide. Replacement of heavily arsenic-contaminated drinking water with low-arsenic water is a potential intervention strategy for arsenosis, although the reversibility of arsenic intoxication has not established. In the present study, we examined urinary excretion of cyclic guanosine 3′,5′-monophosphate (cGMP), a second messenger of the vasoactive effects of nitric oxide, and signs and symptoms for peripheral vascular function in 54 arsenosis patients before and after they were supplied with low-arsenic drinking water in an endemic area of chronic arsenic poisoning in Inner Mongolia, China. The arsenosis patients showed a marked decrease in urinary excretion of cGMP (mean ± SEM: male, 37.0 ± 6.1; female, 37.2 ± 5.4 nmol/mmol creatinine), and a 13-month period of consuming low-arsenic drinking water reversed this trend (male, 68.0 ± 5.6; female, 70.6 ± 3.0 nmol/mmol creatinine) and improved peripheral vascular response to cold stress. Our intervention study indicates that peripheral vascular disease in arsenosis patients can be reversed by exposure cessation and has important implications for the public health approach to arsenic exposure.

The consumption of water contaminated by naturally occurring arsenic poses a serious threat to human health worldwide (Gebel 1999; Nickson et al. 1998). Arsenic causes a wide range of human ailments, including cancer, and vascular diseases such as peripheral and cardiovascular disease, arteriosclerosis, Raynaud’s syndrome, and hypertension (Chen 1990; Engel et al. 1994; Rahman et al. 1999; Wang et al. 2002; Yu et al. 2002). Because the precise mechanisms of arsenic toxicity are still largely undefined, a potentially important remedial action is the termination of further exposure by providing safe drinking water. However, limited data are available on the reversibility of chronic arsenosis in humans.

Our previous studies indicated that chronic exposure to arsenic through drinking water can induce systematic nitric oxide dysfunction, and the impaired NO signaling or bioactivity contributes to arsenic-associated vascular diseases (Pi et al. 2000, 2003; Kumagai and Pi 2004). NO bioactivity is a critical factor in vascular tone, and its impairment can lead to vaso-occlusive diseases (Ganz and Vita 2003). Impaired NO bioactivity contributes, at least in part, to vascular diseases in chronic arsenosis patients (Pi et al. 2000). NO produced endogenously from l-arginine by NO synthases is involved in many physiologic and pathophysiologic processes (Ganz and Vita 2003), and reduced NO production in turn is related to vascular endothelial cell dysfunction (Gewaltig and Kojda 2002). Among the multiple activities of NO, the homeostatic control of the vascular endothelium is directly connected with the activation of soluble guanylate cyclase and the production of cyclic 3′,5′-guanosine monophosphate (cGMP), a critical second messenger of the NO system (Gewaltig and Kojda 2002). Urinary excretion cGMP has been used as a reliable biomarker for endogenous NO production and endothelial cell function (Boger et al. 1997; Cui et al. 2004), and reduced cGMP production is thought to be a biochemical indicator of impaired NO production and peripheral vascular disease (Boger et al. 1997). Therefore, in this follow-up study, we evaluated the impact of intake reduction on chronic arsenic intoxication by investigating urinary excretion of cGMP and peripheral vascular function in arsenosis patients before and after they were supplied with low-arsenic drinking water.

## Methods

### Subjects.

This study was carried out in Gangfangying village, Baotou, Inner Mongolia, China, where high concentrations of arsenic (up to 1,790 μg/L) were present in tube-well water from the end of the 1970s to August 1999. At this point, an alternative community water supply with a drastically lower arsenic level (37 μg/L) was installed. The investigations were conducted in August 1999, just before the new water system was installed, and again in September 2000, 13 months after the switch to less-contaminated drinking water. We obtained informed consent from all participants. Two certified dermatologists and two trained physicians performed physical examinations and administered a standardized questionnaire interview at both time points. A total of 54 volunteer residents (24 males and 30 females, 8–65 years of age; mean age, 36.2 years) who participated in the two surveys in 1999 and 2000 and provided urine samples were enrolled in the present study. Some patients were excluded from this study because of unclear exposure history (14 cases) or because they provided no urine samples in 2000 (10 cases). Before arsenic remediation, 29 of the subjects were identified as having skin lesions typical of arsenosis, which include verrucous hyperkeratoses of the palms and soles of the feet and hypo- and hyperpigmentation in the trunk area (Pi et al. 2002). These symptoms were predominantly found in males (18/29 cases; 62%). In addition, there were 16 reports of cold-weather–associated pain and coldness in the extremities of the feet and hands and/or white fingers, which are regarded as indicators of arsenic-induced peripheral vascular dysfunction. There were no cases of hypertension or overt cardiac dysfunction. We collected fasting peripheral venous blood and morning urine samples for arsenic and/or cGMP analysis. Additionally, for a reference control, we collected 1,132 urine samples from a general population of Japanese men and women (40–65 years of age), living in the two farming communities of Ikawa and Kyowa, known to have minimal exposure to environmental inorganic arsenic.

### Evaluation of arsenic poisoning.

We evaluated peripheral vascular response to cold stress by the difference of finger systolic blood pressure before and after ice-water immersion, which made the surface temperature of the finger decrease by 10ºC. Finger systolic blood pressure was determined by a finger blood pressure monitor (HEM-808F; Omron, Matsusaka, Japan). We measured skin temperature using a Tele-thermometer (WMZ-03, Shanghai Instruments, Shanghai, China).

### Arsenic content in biological samples.

We determined arsenic levels in water and biological samples by atomic absorption spectrophotometry (AA-6800G-ASA-2sp; Shimadzu, Kyoto, Japan) according to our previously reported method (Pi et al. 2003). The detection limit of this method was 1 ng, and the coefficient of variation was < 5%. For standard reference material, we used oyster tissue (no. 1566) from the National Institute of Standards and Technology (Gaithersburg, MD, USA).

### Urinary cGMP level.

We used an ^125^I-labeled cGMP radioimmunoassay kit (Amersham, Buckinghamshire, UK) with a detection limit of 256 fmol/ml to measure urinary cGMP. The interassay coefficient of variation was 3.2% (*n* = 8). To control the differences in renal function, we divided the urinary excretion of cGMP by the urinary creatinine concentration (expressed in nanomoles cGMP per millimoles of creatinine) (Boger et al. 1997). Urinary creatinine levels were determined using a creatinine test kit (Wako Pure Chemical Industries, Osaka, Japan). For each subject, we determined the urinary cGMP excretion level and creatinine twice and used the average value as the final measurement. In the present study, we investigated the effects of arsenic exposure reduction among chronic arsenosis patients based on urinary excretion of cGMP as an indicator of vascular dysfunction. We did not determine the levels of nitrite/nitrate, which are stable NO metabolites, as an alternative index of NO function because several of the tube-wells were contaminated with high levels of nitrite and/or nitrate, which would have distorted these data.

### Statistical analysis.

Data are expressed as mean ± SEM in all cases. Comparisons between data obtained before and after the water switch were performed with a two-tailed, paired Student’s *t*-test. A value of *p* = 0.05 was considered statistically significant.

## Results and Discussion

Table 1 shows that the mean arsenic level in the well water consumed by the 54 subjects from the end of the 1970s to August 1999 (before remediation) was 180 μg/L. In the nearly 20 years of exposure, all of the households enrolled in this study had shared between two and six of these water sources. The arsenic level of the new water supply was 37 μg/L, lower than the World Health Organization (WHO) maximum permissible limit of 50 μg/L for drinking water (WHO 1984). The 13-month exposure reduction markedly decreased the arsenic levels in biological samples, including urine and blood samples, in a sex-independent fashion (Table 1), showing that the new low-arsenic water supply effectively reduced the body burden of arsenic.

**Figure 1:**
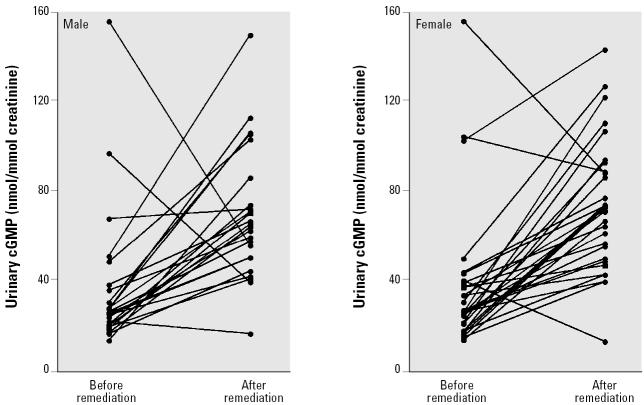
Urinary cGMP excretion in male (*n* = 24) and female (*n* = 30) untreated chronic arsenosis patients before and after the switch to low-arsenic drinking water. Water remediation reversed the arsenic-induced suppression of cGMP production in both males (*p* = 0.0012) and females (*p* < 0.0001).

As shown in Table 2 and Figure 1, the urinary cGMP levels in all age groups of both sexes were significantly depressed before remediation when high levels of arsenic were consumed. This finding was supported by the work of Lee et al. (2003), who reported that arsenite can dramatically inhibit cGMP accumulation in isolated aortic rings of rats. After the 13-month arsenic exposure reduction, urinary cGMP levels increased to normal, as compared to the Japanese general population-based control values of 57.3 ± 2.1 nmol cGMP/mmol of creatinine in males (*n* = 510; 40–65 years of age) and 70.6 ± 3.0 in females (*n* = 622; 40–65 years of age) (Cui et al. 2004).

In agreement with the recovery of the arsenic-induced dysfunction of the NO/cGMP system, as indicated by the increase in urinary cGMP excretion, peripheral vascular response to cold stress, measured as the difference in finger systolic blood pressure before and after ice-water immersion, was significantly improved in male arsenosis patients (Table 3). In female arsenosis patients, although some improvement in finger blood pressure response occurred, it was not statistically significant (Table 3). The difference between peripheral vascular response to cold stress between male and female patients before remediation was also significant (Table 3), which is consistent with more severe exposure in males as evidenced by blood and urinary arsenic (Table 1) and skin lesions. In addition, 3 of the 16 patients reporting cold-weather–associated pain and coldness and/or white fingers showed improvement, although 12 patients had no significant change, and 1 patient became worse (data not shown).

Replacement of drinking water heavily contaminated with arsenic with low-arsenic water is a potential intervention strategy to minimize or reverse the adverse health effects of this toxic inorganic element. Consistent with our previous results (Pi et al. 2000), male and female arsenosis patients in the present study showed a marked depression in urinary excretion of cGMP, and a 13-month period of consuming low-arsenic drinking water reversed this depression. In addition, improved peripheral vascular response to cold stress was clearly observed in male arsenosis patients after consuming low-arsenic water and tended to improve in females. Together, these data suggest that long-term arsenic exposure induces biochemical changes in the vascular system and causes functional vascular damage, which, at least in males, can be reversed by limiting further arsenic intake. Although cGMP production improved, it is possible that females may need a longer period of reduced arsenic exposure for vascular function to be completely restored. In addition, before remediation, males had much higher blood and urinary arsenic levels and showed a higher rate of arsenic-induced skin lesions, indicating more severe intoxication. Thus, improvement of the symptoms of arsenic poisoning may be more readily observed in males.

In conclusion, a 13-month arsenic exposure reduction effectively reverses the arsenic-induced impairment of the NO/cGMP pathway in both males and females and improves peripheral vascular function in males. Additional comprehensive follow-up studies are necessary to document the long-term health benefits of arsenic exposure reduction, but the present results indicate the reduction of arsenic exposure could be an important public health strategy.

## Figures and Tables

**Table 1:** Arsenic levels in drinking water and biological samples before and after drinking water remediation.

	Before remediation		After remediation	
	Males	Females	Males	Females
Biological samples				
Blood (μg/L)*^a^*	9.89 ± 0.21 (*n* = 22)	6.10 ± 0.79 (*n* = 23)	2.52 ± 0.23* (*n* = 22)	1.83 ± 0.19* (*n* = 23)
Urine (μg/g Cr)	424.5 ± 122.9 (*n* = 24)	292.5 ± 66.6 (*n* = 30)	177.2 ± 37.7* (*n* = 24)	161.5 ± 32.7* (*n* = 30)
Drinking water (μg/L)	180 ± 60		38	

Cr, creatinine. Data expressed as mean ± SEM. Before remediation, *n* = 37 community wells; after remediation, *n* = 1 low-arsenic community well.

a Nine blood samples were not available.

* Significantly reduced (*p* < 0.05) compared with appropriate sex-matched population values from before remediation.

**Table 2:** Urinary cGMP excretion (nmol/mmol creatinine) before and after switching to low-arsenic drinking water.

	Before remediation		After remediation	
Age (years)*^a^*	Males	Females	Males	Females
8–13	22.1 ± 3.0 (*n* = 3)	27.2 ± 1.7 (*n* = 4)	73.7 ± 19.8* (*n* = 3)	66.8 ± 22.4* (*n* = 4)
21–40	29.6 ± 3.6 (*n* = 6)	33.3 ± 5.5 (*n* = 15)	60.1 ± 3.1* (*n* = 6)	76.8 ± 7.8* (*n* = 15)
41–65	45.1 ± 10.2 (*n* = 15)	42.9 ± 11.1 (*n* = 11)	67.2 ± 6.5* (*n* = 15)	69.9 ± 8.5* (*n* = 11)

Data expressed as mean ± SEM.

a Age in August 1999.

* Significantly different (*p* < 0.05) from measurement taken in August 1999, immediately before the introduction of low-arsenic drinking water.

**Table 3:** Peripheral vascular response to cold stress before and after switching to low-arsenic drinking water.

Sex	No.	Before remediation	After remediation
Males	15	41.5 ± 5.8	26.0 ± 4.8*
Females	16	28.6 ± 3.4**	22.6 ± 4.3

Data expressed as mean ± SEM (mmHg).

* Significantly different (*p* < 0.05) from measurement taken before remediation.

** Significantly different (*p* < 0.05) from males before remediation.
